# Might Routine Vitamin A Monitoring in Cystic Fibrosis Patients Reduce Virus-Mediated Lung Pathology?

**DOI:** 10.3389/fimmu.2021.704391

**Published:** 2021-11-09

**Authors:** Robert E. Sealy, Sherri L. Surman, Peter Vogel, Julia L. Hurwitz

**Affiliations:** ^1^ Department of Infectious Diseases, St. Jude Children’s Research Hospital, Memphis, TN, United States; ^2^ Department of Pathology, St. Jude Children’s Research Hospital, Memphis, TN, United States; ^3^ Department of Microbiology, Immunology, and Biochemistry, University of Tennessee Health Science Center (UTHSC), Memphis, TN, United States

**Keywords:** cystic fibrosis, vitamin A, respiratory virus infection, mouse model, prevention

## Abstract

Cystic fibrosis (CF) is an autosomal recessive gene disorder that affects tens of thousands of patients worldwide. Individuals with CF often succumb to progressive lung disease and respiratory failure following recurrent infections with bacteria. Viral infections can also damage the lungs and heighten the CF patient’s susceptibility to bacterial infections and long-term sequelae. Vitamin A is a key nutrient important for immune health and epithelial cell integrity, but there is currently no consensus as to whether vitamin A should be monitored in CF patients. Here we evaluate previous literature and present results from a CF mouse model, showing that oral vitamin A supplements significantly reduce lung lesions that would otherwise persist for 5-6 weeks post-virus exposure. Based on these results, we encourage continued research and suggest that programs for the routine monitoring and regulation of vitamin A levels may help reduce virus-induced lung pathology in CF patients.

## Cystic Fibrosis (CF), the Disease

CF is a recessive genetic disorder that affects tens of thousands of patients globally. The disease is due to mutations in the cystic fibrosis transmembrane conductance regulator gene (*CFTR*) that encodes an ion channel protein. Mutations result in abnormal epithelial fluid transport in respiratory and digestive tracts (1). Individuals with CF often succumb to progressive lung disease and respiratory failure following recurrent infections with bacteria including *Staphylococcus aureus, Haemophilus influenza, and Pseudomonas aeruginosa* (2, 3). Although some reports suggest that CF patients clear viruses as well as their healthy counterparts (4), virus infections can damage the lung, increase susceptibility to bacterial infections, and worsen outcomes. CF patients with respiratory viral infections often have culture-positive bacterial pathogens (5, 6). In one study, viral respiratory infections were evident among 65% of exacerbations in patients with CF (7, 8). These data highlight the importance of virus control as an integral component of CF patient care.

## Vitamin A

Vitamin A is an essential nutrient, necessary for healthy immune responses against respiratory pathogens, control of infectious diseases, and the integrity of mucosal epithelial cells. Vitamin A effector functions occur at the cell surface, within the cell cytoplasm, and within the nucleus of target cells (9–11). Experiments in animal models have shown that when vitamin A is low, host susceptibility to pathogens is increased (12–15). In vitamin A deficient animals, vaccine responses are weak, and serious outcomes follow viral infections and bacterial co-infections (15–17). After clearance of replicating pathogens, residual foreign antigens and consequent inflammatory responses may persist (18).

## Vitamin A Deficiencies/Insufficiencies Are a Global Concern

It is noteworthy that vitamin A deficiency/insufficiency, a condition once presumed to affect developing countries only, is now frequent in the developed world including the United States (19, 20). A unique concern is raised for the CF patient due to pancreatic insufficiency and malabsorption (21–23). Some centers report that 10-40% of their CF patients are vitamin A deficient (VAD) (24). Vitamin A monitoring is sometimes performed as a routine and supplementation is sometimes used as a treatment option in deficient patients (25, 26). Sapiejka et al. observed higher blood vitamin A levels in patients who received vitamin A supplements. They also found that low FEV1 levels (<80%) were significantly more frequent in patients with vitamin A levels <300 ng/ml.

Unfortunately, health guidelines vary (24–31) and many clinical centers overlook vitamin A. Even when serum vitamin A levels appear normal in an individual (32), there may be inadequate vitamin A levels in parenchymal tissues (33). Tissue deficits can be missed in humans when standard blood tests are employed and may be revealed by vitamin A dose response tests (34). When vitamin A levels are insufficient/deficient in an animal, whether due to a nutritional deficiency or an underlying health condition, vitamin supplements can improve pathogen-specific immune responses and pathogen control (13, 17, 35, 36).

## Experiments in a CF Mouse Model Demonstrate That Persistent Lung Pathology Following a Respiratory Virus Infection Is Reduced by Oral Vitamin A Supplements

We used a CF mouse model to ask if vitamin A supplements could reduce virus-induced lung pathology that persists after a parainfluenza virus respiratory infection. Experiments were with *Cftr^tm1Unc^* Tg(FABPCFTR)1Jaw mice that were purchased from Jackson Laboratories (Bar Harbor, Maine) or bred from Jackson mice at St Jude Children’s Research Hospital. *Cftr^tm1Unc^* Tg(FABPCFTR)1Jaw mice are homozygous for a mutation in the mouse *Cftr* gene. The targeted knock-out allele (*Cftr^tm1Unc^*) was originally created by insertion of a neomycin selection cassette into the gene at sequences corresponding to codon 489. Mice were additionally homozygous for a functional human *CFTR* gene expressed under the control of the rat intestinal fatty acid-binding protein 2 (*Fabp2*) gene promoter (FABP-hCFTR) (37, 38). The latter gene was necessary to rescue protein function in the mouse intestinal epithelium, to correct malabsorption. Otherwise, mice did not survive to adulthood. We note that in humans with CF, malabsorption is often corrected using pancreatic enzyme replacement therapy (39). Mice were termed ‘CF’ for simplicity. A preliminary test of serum retinol binding protein (RBP), a surrogate for serum vitamin A (retinol), showed no significant differences between mice carrying the CFTR mutation and controls.

Experiments were designed and performed in triplicate to test the benefits of vitamin A supplements for reduction of residual lung pathology that persisted several weeks after a viral infection in CF animals. In each of three experiments, there were two groups of mice with 7-8 mice per group. Both groups were infected with parainfluenza virus (Sendai virus, SeV). Test CF mice, but not controls, were additionally treated with oral doses of vitamin A on days -7, -3, 0, and +3 relative to the intranasal infection. The extent and severity of residual pulmonary lesions were then assessed at 5-6 weeks post-infection, a time after virus had been cleared (no residual viral antigen was detected by immunohistochemical staining at that time), to identify persistent consequences of a virus infection in the CF model.

**Figure 1:**
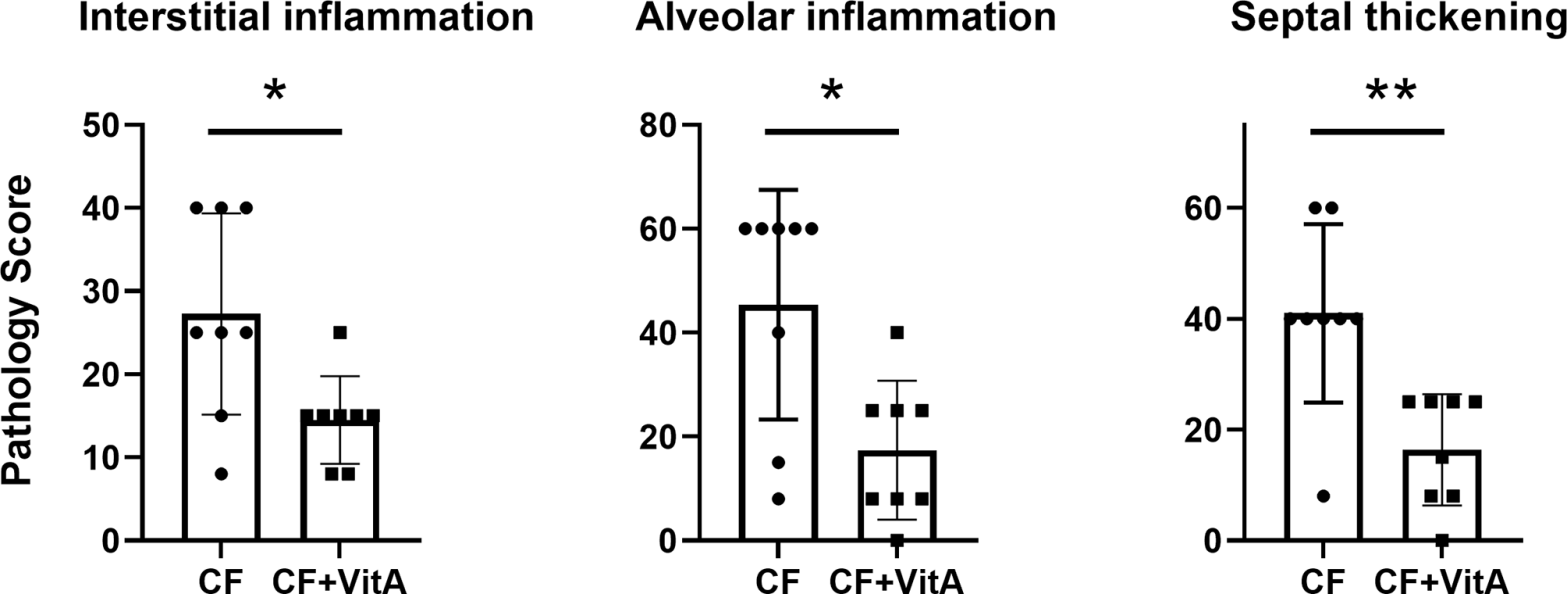
Vitamin A supplements at the time of parainfluenza virus infections reduce lung lesions scored 5-6 weeks post-infection. Data are from one of three experiments demonstrating the reduction of lung lesions in CF mice supplemented with vitamin A at the time of a virus infection. Lesions were given relative scores. Each symbol represents a different test mouse (no lesions were observed in control, uninfected mice). In each of the three experiments, the vitamin A-supplemented mice (CF+VitA) exhibited significantly or marginally reduced scores for interstitial inflammation, alveolar inflammation, and septal thickening at weeks 5-6 post-virus infection compared to mice that received no supplement (CF). Means with standard deviations are shown. Mann Whitney tests were performed to compare groups (*p < .05, **p < .01).

After SeV infections of CF mice, vitamin A-supplemented mice exhibited fewer lung lesions at 5-6 weeks compared to unsupplemented mice. In **Figure 1**, data are shown from one experiment. The three types of lesions represented in this Figure (interstitial inflammation, alveolar inflammation, and septal thickening) were: (a) reduced in supplemented mice compared to controls in each of the three experiments, and (b) significantly reduced in supplemented mice compared to controls in at least one of the three experiments.

**Figure 2:**
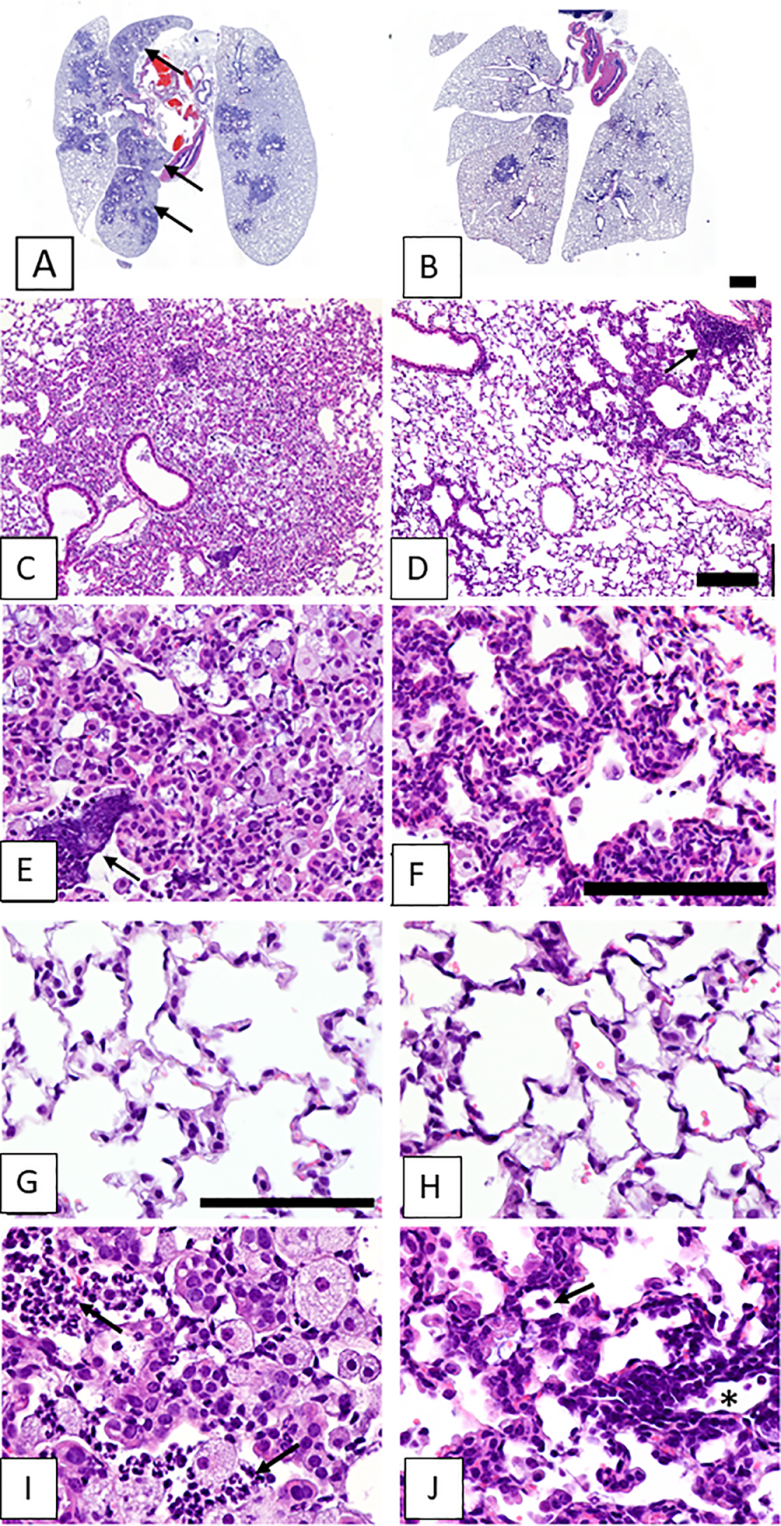
Lung lesions from vitamin-unsupplemented and supplemented in CF mice 5-6 weeks post-parainfluenza virus infections. Images represent lung tissues sampled 5-6 weeks after an SeV infection. Images in the left column were taken when mice received no vitamin A supplements. Images in the left column represent a mouse that received no vitamin A supplements. Images in the right column represent a mouse that received vitamin A supplements at the time of SeV infections. Panel **(A)** shows lesions from an unsupplemented mouse. These frequently extended from terminal airways to the lung margins and coalesced with lesions surrounding other bronchioles to involve entire lung lobes (arrows). **(C)** Lesions were characterized by indistinct borders and alveoli filled with debris and cellular infiltrates. **(E)** Cell infiltrates consisted mostly of large (activated) alveolar macrophages, with scattered clusters of neutrophils, both intact and degenerating (arrow). Panel **(B)** shows Pulmonary lesions from a mouse taken 5-6 weeks after an SeV infection with vitamin A supplementation. Lesions were small and sharply demarcated, rarely reaching the lung margin and not affecting entire lung lobes. **(D)** At higher magnification, lesions were observed with generally clear alveolar spaces, sharply demarcated from surrounding normal parenchyma. Peribronchiolar lymphoid aggregates were also evident (arrow). **(F)** Septal thickening in the lesioned area was due to interstitial inflammatory cell infiltrates (lymphocytic) and diffuse hypertrophy of alveolar epithelium. **(G, H)** Images include areas of normal tissue for comparison to areas of diseased tissue. Images were from unsupplemented and supplemented mice, respectively. **(I, J)** Images show lesioned areas at 60X magnification in unsupplemented and supplemented mice, respectively. **(I)** a virus-damaged area in which alveoli were filled with clusters of neutrophils (arrows) and large foamy alveolar macrophages (alveolar inflammation). **(J)** Pulmonary lesions were characterized by air-filled alveoli with septa thickened by hypertrophic alveolar epithelium and interstitial lymphocytic infiltrates (interstitial inflammation). Lymphocytic infiltrates were prominent surrounding blood vessels (near asterisk). Open alveolar spaces contained normal-sized alveolar macrophages with relatively small amounts of cytoplasm (arrow). Scale bars: B = 1 mm; D = 200 μm; F, G = 100 μm.

**Figure 2** shows images of lung tissues observed 5-6 weeks after SeV infections in CF mice that did not receive (left column) or did receive (right column) vitamin A supplements. In **Figure 2A** are shown lesions from an unsupplemented mouse that extended from terminal airways to the lung margins, sometimes coalescing with lesions surrounding other bronchioles to involve entire lung lobes. Higher magnification images revealed lesions with indistinct borders and alveoli filled with debris and cellular infiltrates (**Figure 2C**). These cell infiltrates consisted mostly of large (activated) alveolar macrophages, with scattered clusters of neutrophils, both intact and degenerating (see arrow, **Figure 2E**). In contrast, the lesions in a CF mouse that received vitamin A supplements at the time of SeV infection were sharply demarcated and relatively small, rarely reaching the lung margin and generally only partially surrounding terminal airways (**Figure 2B**). Higher magnification images revealed thickened alveolar septa and predominantly clear alveolar spaces with relatively little cellular debris and few alveolar macrophages (**Figures 2D, F**). Peribronchiolar lymphoid aggregates were observed (see arrow, **Figure 2D**). Septal thickening involved interstitial inflammatory cell infiltrates (lymphocytic) and diffuse hypertrophy of alveolar epithelium (**Figure 2F**). Images from normal areas of tissue that were unaffected by virus are shown in **Figures 2G, H** from an unsupplemented and supplemented mouse, respectively. Here, there were no notable differences between alveoli. **Figures 2I, J** provide the highest (60X) magnifications of inflamed virus-damaged areas from an unsupplemented and supplemented mouse, respectively. In the unsupplemented mouse (**Figure 2I**), as described above, alveoli were filled with clusters of neutrophils (arrows) and large foamy alveolar macrophages (alveolar inflammation). In contrast, in the vitamin A-supplemented mouse (**Figure 2J**), smaller pulmonary lesions at 5-6 weeks after SeV infection were characterized by air-filled alveoli with septa thickened by hypertrophic alveolar epithelium and interstitial lymphocytic infiltrates (interstitial inflammation). Lymphocytic infiltrates were especially prominent surrounding blood vessels (near asterisk). The open alveolar spaces contained normal-sized alveolar macrophages with relatively small amounts of cytoplasm (arrow).

This study had limitations in that it used a single CF mouse model and focused on a single parainfluenza virus infection and a single vitamin A dosing regimen. The mechanism by which vitamin A protected animals from virus-induced pathology was not defined in this study. Studies were performed after virus had been cleared and when viral antigens were no longer detected by immunohistochemical studies. Possibly, the employment of a more sensitive assay such as the polymerase chain reaction (40) or a T-cell based assay (18) would have detected residual nucleic acids or peptide fragments pertinent to long-term health outcomes. Follow-up studies are warranted to define mechanisms by which vitamin A protected the animals. Experiments may include different CF models (41–43), different pathogens, and different vitamin A dosing regimens with comprehensive measurements of virus titers, inflammatory cell subsets in the lung (aided by flow cytometry), vitamin levels (in blood, liver, and lung), animal weights, and immune functions throughout the time course.

## Why Might Vitamin A Supplements reduce Lung Lesions Post-Virus Infection?

Vitamin A is pleiotropic in function, affecting numerous cell types including B cells, T cells, dendritic cells (DCs) and respiratory tract epithelial cells. As an example, vitamin A influences the expression of α4β7 and CD103, a component of the αEβ7 homing receptor, on T cells and DCs. These homing receptors regulate adherence and motility of immune cells, important for rapid virus-specific immune responses (44–47). As a second example, vitamin A assists B cell production of IgA, a key effector for the local prevention of respiratory tract infections (35, 48–50). In some systems, vitamin A has been associated with the induction of interferon (IFN) α/β and the support of IFN functions (51–53). By enhancing adaptive and innate immune functions, vitamin A may improve viral clearance and thereby prevent lung damage.

There are a multitude of additional effects (both immunostimulatory and immunoregulatory) of vitamin A on pathogen control including influences on myeloid cell expansion (54) and Treg or Th17 maturation [with variable effects *in vitro* and *in vivo* (12)]. Mechanisms and cause-effect relationships are complex in that an influence of vitamin A on one cell type (e.g., upregulation of B cell activity) may have numerous downstream influences (e.g., clearance of antigen and reduction of cytokines) (12, 17, 18, 55). Disease consequences can, in turn, influence levels of RBP (an acute-phase protein and an important blood escort for retinol) and vitamin A.

Apart from affecting immune responses, vitamin A also supports the growth and integrity of epithelial cells (14), features associated with wound prevention and repair. When introduced into *in vitro* systems, vitamin A assists the establishment of epithelial monolayers and organ culture systems (56). Epithelial cells of the respiratory tract express retinaldehyde dehydrogenase 2 (RALDH2 or ALDH1A2), an enzyme necessary for conversion of retinaldehyde to its end-stage metabolite, retinoic acid (48).

Our previous studies have shown that VAD associates with delayed clearance of respiratory viruses and viral antigens (15, 18). Vitamin A supplements can improve vitamin A levels in peripheral tissues, including the lung, and improve immune-mediated clearance of respiratory viral infections (12, 13, 35, 36). Consequently, epithelial barriers are maintained and antigen-driven inflammatory responses are resolved (18). Altogether, the benefits of vitamin A are clearly multi-factorial and include support of (i) rapid immune function, (ii) rapid clearance of virus and viral antigens, and (iii) epithelial growth, to prevent lung pathology and improve clinical health.

## Should Vitamin A Supplements Be Used Indiscriminately in CF Patients?

There is unfortunately no consensus guideline for vitamin A monitoring and control in CF patients. Replete vitamin A levels are best attained by maintenance of healthy diets, but supplementation programs provide a back-up solution when patients repeatedly suffer malnutrition. Some centers encourage vitamin A supplementation, while others do not (24, 27–30). Indiscriminate use of vitamin A supplements is sometimes advocated, but the fine-tuning of vitamin supplementation programs may yield a better result. This is because vitamin A supplements, particularly if administered at high doses, can cause harm (57). As an example, Bresee et al. showed that when vitamin A supplements were administered to children who were hospitalized with respiratory syncytial virus (RSV) infections, the supplemented children experienced longer hospital stays than placebo controls (58). In our study of influenza vaccine immunogenicity in children, a supplement with vitamin A+D improved responses in children who were vitamin A+D insufficient or deficient at baseline, but reduced responses in children who were vitamin replete (20). Baseline vitamin levels [and levels of other nuclear receptor ligands that may cross-regulate vitamin functions (10, 59)] will clearly influence outcome.

Few controlled clinical studies of vitamin A supplementation in CF patients have been performed (25, 60). In one small study of CF patients, high-dose beta-carotene supplementation was compared to placebo and was found to reduce requirements for antibiotics during a three-month period (25), demonstrating a prophylactic benefit. Additional, controlled clinical trials are needed to determine precisely when vitamin A supplements provide benefit and what characteristics (e.g., age, obesity, sex, and baseline vitamin levels) associate with the best outcome among CF patients.

## Conclusion

In a mouse model for CF, vitamin A supplements were shown to improve lung integrity 5-6 weeks after a respiratory viral infection. Results encourage additional basic research and the performance of new clinical trials to determine when diet modifications and/or vitamin A supplements are beneficial to this patient population. Our perspective is that vitamin A should be routinely monitored and regulated in CF patients to improve lung health and protect from persistent tissue damage caused by infectious disease.

## Methods

Experiments were reviewed and approved by the IACUC of St. Jude Children’s Research Hospital (St. Jude). All experiments were with *Cftr^tm1Unc^* Tg(FABPCFTR)1Jaw mice that were purchased from Jackson Laboratories (Bar Harbor, Maine) or bred from Jackson mice at St Jude. To breed mice at St. Jude, females that were heterozygous for the mouse *Cftr^tm1Unc^* Tg and homozygous for FABP-hCFTR were crossed to males that were homozygous for *Cftr^tm1Unc^* Tg and homozygous for FABP-hCFTR. Progeny were genotyped at Transnetyx (Cordova, Tennessee) and mice that were homozygous for both *Cftr^tm1Unc^* Tg and FABP-hCFTR were selected for study. Mice were anesthetized with isoflurane and infected intranasally with 250 plaque forming units (pfu) of Sendai virus (SeV, a mouse parainfluenza virus-type 1) in 30 μl phosphate buffered saline. Test mice were supplemented with vitamin A (retinyl palmitate, 600 IU/mouse, Nutrisorb A, Interplexus Inc. [Kent, WA] in 100 μl PBS) by oral gavage on days -7, -3, 0, and +3 relative to infection. The route of administration and dose were selected to test a simple, clinically translatable, prophylactic methodology. Mice were rested for 5-6 weeks post-infection and then euthanized with CO_2_. The lungs were infused with formalin, fixed by immersion in 10% neutral buffered formalin for several weeks, embedded in paraffin, sectioned, and stained with hematoxylin and eosin stains. Slides were stained for residual viral antigens (18), but scored negative. Slides were analyzed and scored by a veterinary pathologist who was blinded to mouse groups at the time of analyses in the Veterinary Pathology Core Department at St. Jude. Scores were given for interstitial inflammation, alveolar inflammation, alveolar protein/fibrin, septal thickening, lymphoid nodules, epithelial hyperplasia, bronchiolization, and fibrosis. Each of these types of pulmonary lesions was assigned a severity grade on a 1-5 ranked scale as follows: 0 = no lesions (no changes outside of normal limits; 0% tissue affected); 1 = minimal, focal to multifocal, inconspicuous (rare lesions, barely above normal limits; <5% tissue affected); 2 = mild, multifocal, prominent (small, widely separated or focal lesions, limited severity; <10% tissue affected); 3 = moderate, multifocal, prominent; 4 = marked, multifocal or coalescing, lobar (>60% tissue affected); 5 = severe, extensive or diffuse, multilobar, with consolidation (>80% tissue affected). Intermediate severity grades were also assigned as needed. Grades were then converted to weighted semi-quantitative scores to better reflect the extent of damage and lung involvement as follows: 0 = 0; 1 = 1; 1.5 = 8; 2 = 15; 2.5 = 25; 3 = 40; 3.5 = 60; 4 = 80; 4.5 = 90; 5 = 100. Semiquantitative scores are shown for each animal on Y axes. Experiments were performed in triplicate with 7-8 mice per group in each experiment.

## Data Availability Statement

The raw data supporting the conclusions of this article will be made available by the authors, without undue reservation.

## Ethics Statement

The animal study was reviewed and approved by the IACUC at St. Jude.

## Author Contributions

All authors contributed to the design, methodology, and interpretation of experiments. JLH authored the first draft of the paper and all authors reviewed and edited the paper. All authors contributed to the article and approved the submitted version.

## Funding

Research was supported, in part, by NIH NCI P30CA21765 and ALSAC.

## Conflict of Interest

The authors declare that the research was conducted in the absence of any commercial or financial relationships that could be construed as a potential conflict of interest.

## Publisher’s Note

All claims expressed in this article are solely those of the authors and do not necessarily represent those of their affiliated organizations, or those of the publisher, the editors and the reviewers. Any product that may be evaluated in this article, or claim that may be made by its manufacturer, is not guaranteed or endorsed by the publisher.
